# Light-coupled cryogenic probes to detect low-micromolar samples and allow for an automated NMR platform

**DOI:** 10.5194/mr-5-61-2024

**Published:** 2024-05-15

**Authors:** Wolf Wüster, Pit Gebbers, Alois Renn, Matthias Bütikofer, Sophie Rüdiger, Roland P. Riek, Felix Torres

**Affiliations:** 1 Zurich University of Applied Sciences (ZHAW), Institute of Applied Mathematics and Physics, Technikumstrasse 9, 8400 Winterthur, Switzerland; 2 Federal Institute of Technology Zurich (ETHZ), Institute of Molecular Physical Science, Vladimir-Prelog-Weg 2, 8093, Zürich, Switzerland; 3 NexMR AG, Wiesenstrasse 10A, 8952, Schlieren, Switzerland

## Abstract

Recent advances in NMR fragment screening use sample illumination to boost NMR sensitivity, reduce measurement time to a few seconds, and reduce sample concentration to a few micromolars. Nevertheless, the absence of a fully automated solution to measure several hundreds of samples with photoinduced hyperpolarization limits the large-scale applicability of the method. We present a setup to couple an optical fiber with a cryogenic probe using the flow-cell accessory port. This setup is compatible with commercially available autosamplers, enabling the fully automated measurement of several hundreds of samples per day.

## 1 Introduction

Photochemically induced dynamic nuclear polarization (photo-CIDNP) enhances NMR sensitivity thanks to the radical pair recombination after nuclear spin-dependent singlet–triplet mixing in a magnetic field (Ward and Lawler, 1967; Bargon and Fischer, 1967; Kaptein and Oosterhoff, 1969; Closs, 1969). Typically, a photosensitizer, e.g., fluorescein or Atto Thio 12, is excited with light to generate the radical pair with a small molecule of interest, e.g., tryptophan, tyrosine, histidine, or diverse heteroaromatic scaffolds (Okuno and Cavagnero, 2016; Morozova and Ivanov, 2019; Hore and Broadhurst, 1993; Torres et al., 2023, 2021a, b). After recombination, the nuclear spin population of the small molecules is out of Boltzmann equilibrium, yielding signal-to-noise enhancement (SNE) in the range of 20 to 100-fold, depending on the photosensitizer–molecule pair, its magnetic parameters, g-factors, the hyperfine couplings, and the magnetic field (Torres et al., 2021a, b; Sobol et al., 2019). Photo-CIDNP is typically performed in an aqueous buffer at room temperature and requires a few seconds of light irradiation. Therefore, it is an ideal hyperpolarization method for biological application, including fragment screening, where thousands of fragment molecules are screened against a biological target to identify an interaction. Photo-CIDNP screening of fragment libraries was reported with an experimental time of a few seconds and low micromolar concentration at high and low magnetic fields (Torres et al., 2023; Stadler et al., 2023). Other applications potentially requiring high throughput were described recently, such as metabolomics (Kuhn et al., 2024). However, the sample illumination requires the insertion of an optical fiber into the NMR tube, impeding the use of automatic sampling machines, which would break the fiber. This drawback is major as the sample then needs to be inserted by hand, reducing the throughput and monopolizing the staff's time over a repetitive task. While a flow-through NMR where a high‐performance liquid chromatography (HPLC) system is in fluidic connection with a flow cell integrating an optical fiber was designed to solve this issue (Torres et al., 2023), this solution presents the disadvantage of regular clogging due to the accumulation of small molecules or proteins in micrometer-sized tubing. NMRtorch is an alternative option to illuminate the sample with NMR tubes without an optical fiber, particularly in the case of high optical density due to the dye present in diverse light-coupled NMR applications comprising photo-CIDNP (Bramham and Golovanov, 2022). Nevertheless, integrating NMRtorch to commercially available autosamplers is not straightforward and would require hardware adaptation.

Furthermore, the samples measured for fragment screening contain a relatively low concentration of photosensitizer (2–10 µ M) (Torres et al., 2023; Stadler et al., 2023), and the optical density is not critical to obtain sufficiently high SNE. Previous designs from others inspired this work to irradiate the NMR tubes from the bottom. These previous experiments integrated light with quartz rods or optical mirrors into room temperature probes, and they use flat-bottomed NMR tubes to improve the coupling of the light into the sample (Kuhn, 2013; Kuprov and Hore, 2004). More recent work uses optical fibers inserted into drilled room temperature probes and modified NMR tubes (Tolstoy et al., 2009; Koeppe et al., 2011). The objective of the present work is to demonstrate that it is possible to integrate an optical fiber without any hardware modification, which is more modular and can be inserted into a cryogenic probe using the flow-cell accessory port. This presents the advantage of the benefit from the state-of-the-art performances of cryogenic probes, and it is easily installed using off-the-shelf and affordable laser components. Finally, we show that it is possible to take advantage of the spherical shape of the bottoms of standard NMR tubes and the resulting lensing effect to achieve sufficient light–sample coupling. The platform presented in this article is compatible with fully automated solutions such as commercially available autosamplers. It uses standard NMR consumables, guaranteeing a straightforward implementation for the photo-CIDNP NMR field.

## 2 Results and discussion

**Figure 1:**
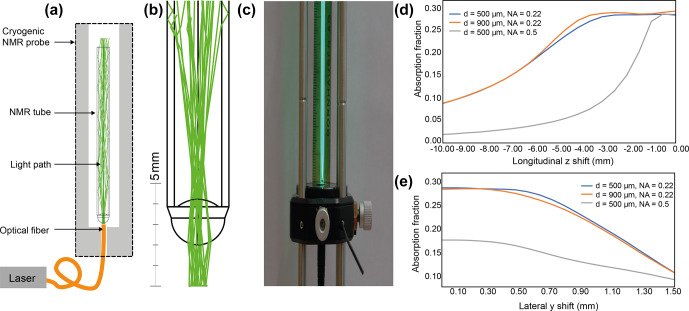
**(a)** Sketch of the optical excitation scheme. The excitation laser is coupled to a multimode fiber. The fiber is inserted at the bottom of the cryogenic probe and is brought into the vicinity (distance of a few mm) of the NMR sample tube. **(b)** Ray-tracing simulation of the light path. The method relies on the lensing effect of the standard NMR tube bottom. The light is confined within the NMR sample volume and NMR tube glass via total internal reflection. **(c)** Optical benchtop experiment with a multimode fiber in contact with a 3 mm diameter NMR tube. The light path is visible via the fluorescence of the fluorescein sample. **(d)** Simulation of the absorbed power (AU) in the NMR sample volume as a function of longitudinal axial distance (longitudinal z shift) for different NA values and optical fiber diameters (500 and 900 µ m). **(e)** Simulation of the absorbed power (AU) in the NMR sample volume as a function of lateral axial distance (lateral y shift) for different NA values and optical fiber diameters (500 and 900 µ m). The absorption fraction is the light flux absorbed within the NMR-active sample volume divided by the light flux incident on the tube bottom. The absorption is computed by the ray-tracing software using the Beer–Lambert law, assuming a fluorescein concentration of 10 µ M L ${}^{-1}$ (0.10752 cm ${}^{-1}$ ).

We use the flow-cell accessory port of a cryogenic probe to insert an optical multimode fiber in a non-invasive way (Fig. S1 in the Supplement). The full functionality of the cryogenic probe is always granted while the fiber is inserted. We take advantage of the fact that cryogenic probes are designed to be operated with flow-cell tubes inserted at the bottom of the cryogenic probe. We find that an optical multimode fiber with a maximum outer diameter of roughly 1 mm can be used instead of a flow tube. The fiber can be inserted into the cryogenic probe and brought into the NMR tube's proximity without constriction. The illumination scheme is depicted in Fig. 1a. The multimode fiber is inserted until it reaches the lower end of the NMR tube. The light emerges from the optical fiber as a light cone whose divergence angle depends on the fiber's numerical aperture (NA). The light cone illuminates the bottom of the NMR sample and is refracted into the sample due to the refractive index difference of air ( n=1 ) and the borosilicate glass of the NMR sample tube ( n=1.47 ). In the following, we describe the results for 3 mm tubes as they are.

Due to the spherical shape of the bottom of the NMR tube, the light is transmitted into the sample liquid (Fig. 1b). The shape of the tube bottom can be described as a spherical convex lens with two different curvatures. For a standard 3 mm NMR tube, we find an outer curvature of 1.3 mm, an inner curvature of 1.6 mm, and a tube wall thickness of 0.35 mm (Fig. S2). We simulated the light path via optical ray tracing (Fig. 1a and b). For this, the software package Ansys Zemax OpticStudio was used in non-sequential mode. The light is confined to the liquid sample and the tube walls via total internal reflection, resulting from the difference in the refractive index of air ( n=1.00 ) and the sample, which is essentially water ( n=1.33 ). Light rays that travel in the liquid at a maximum angle of 41.3° with respect to the sample wall will be confined within the sample and the tube walls. The confinement of the light within the sample was verified experimentally by bringing a multimode fiber close to an NMR sample tube filled with fluorescein (Fig. 1c).

As the light emerges as a cone from the optical fiber output, the effect of the distance between the optical fiber output and the bottom of the tube was evaluated. As a result of the lensing effect of the bottom of the NMR tube, there are different scenarios depending on the position of the optical fiber output relative to the focal point of the NMR tube's bottom (approximately 2 mm). With a distance of 0–2 mm, the light beam is slightly divergent within the sample liquid; with a distance greater than 2 mm, the light beam is focused within the NMR sample (Fig. S3). In agreement with our postulate that the NMR tube bottom is a lens, the light path is collimated when the distance between the optical fiber output and the NMR tube's bottom matches the focal point, i.e., approximately 2 mm (Fig. S3). This agrees with the back focal length of 2.1 mm computed using the thick-lens formula (Greivenkamp, 2004) and the values shown in Fig. S2. The absorbed light power in the NMR-active sample region is stable for a distance range of 0.0 to 4.0 mm and starts decreasing for distances greater than this (Fig. 1d). While the diameter of the optical fiber seems to not affect the coupling, we observe that the NA plays a critical role, and only NA = 0.22 allows a robust setup (Fig. 1d).

The light path inside the sample volume depends on the exact positioning of the optical fiber output to the sample. By mechanical construction of the cryogenic probe flow accessory port, the fiber is coaxial to the sample tube. Nevertheless, since the fiber is flexible and does not fill the port fully, the alignment is expected to be imperfect, translating into a transverse offset. The sensitivity of the optical light path to transverse offset was evaluated for an optical fiber of 500 µ m and NA = 0.22 and a 3 mm NMR sample tube, using ray-tracing simulations (Fig. S4). The optimal position is found where the fiber is coaxially centered with a distance to the tube on the order of 2 mm. In this case, the light beam is more or less collimated within the sample liquid. When a transverse offset of 0.5 mm is applied, the absorbed fraction reduces by 3 % (and 30 % for a transverse offset of 1 mm) (Fig. 1e). Moreover, the light is confined to the NMR tube by the higher refractive index of the tube ( n=1.47 ) compared to the air ( n=1.00 ). Considering that the typical multimode optical fiber diameter is 0.4–0.9 mm and the accessory port of the cryogenic probe is approximately 1.2 mm, an offset above 0.8 mm is unlikely. Therefore, even if the fiber position is offset, the light path is altered, but most of the light remains within the sample volume due to total internal reflection (Fig. S4). In summary, these results support our experimental findings that inserting the optical fiber near the NMR tube yields illumination of the sample in the NMR tube.

**Figure 2:**
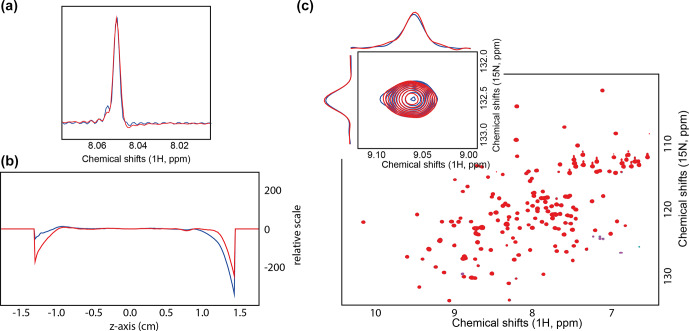
**(a)** Overlaid 1D 1H spectrum of 0.3 % CHCl ${}_{3}$ in acetone-d ${}_{6}$ . The spectrum was recorded with 32 000 points, i.e., 1 s acquisition. **(b)** Field map recorded after shimming with (blue) and without optical fiber from the bottom (red). **(c)** Overlaid [ ${}^{15}$ N, ${}^{1}$ H]-HSQC spectra of the KRAS G13D mutant with (blue) and without optical fiber from the bottom (red).

To verify the absence of field homogeneity perturbation caused by inserting an optical fiber close to the $B_{1}$ coils, we recorded the spectra of 0.3 % CHCl ${}_{3}$ in acetone-d ${}_{6}$ , the standard line shape reference sample (Fig. 2a). The line shape did not exhibit any particular difference between the two setups; as shown in Table 1, the linewidths at different levels from the maximum peak intensity are similar. The linewidth was also measured using the *peakw* command, resulting in two identical values of 1.147 Hz at 66 % of the peak maximum.

**Table 1:** Linewidths of the CHCl ${}_{3}$ peak for different setups, including an optical fiber in the cryogenic probe or not.

Setup	Linewidth at 0.11 %	Linewidth at 0.55 %	Linewidth at 50 %
	maximum (Hz)	maximum (Hz)	maximum (Hz)
No fiber	4.0	3.6	1.23
Fiber	4.3	3.7	1.23

Finally, the field map was recorded after shimming (*topshim map* command in Topspin^®^), as shown in Fig. 2b. The extrema regions, i.e., between 1.0 and 1.5 cm, respectively -1.0 and -1.5 cm, show differences in the measured field, which is unrelated to the optical fiber insertion as they appear at both ends. These differences are minor and result from how the algorithm fits the best polynomial in the area where the sample is measured ( -0.5 to 0.5 cm). The similarity in shimming performances is reflected in the topshim reports with a $B_{0}$ standard deviation of 0.24 and 0.22 Hz (*topshim report* command in Topspin^®^) for the setups with and without fiber, respectively. This positive outcome was expected as prior work from other groups did not report field homogeneity or shimmability issues (Tolstoy et al., 2009; Koeppe et al., 2011); nevertheless, prior designs were for room temperature probes and not cryogenic probes. Therefore, it was essential to validate the compatibility of this setup for a different probe geometry.

To exemplify the performances of the fiber-coupled cryogenic probe, we recorded the most emblematic experiment of bioNMR, namely, a [ ${}^{15}$ N, ${}^{1}$ H]-hetero single-quantum coherence (HSQC) (Bodenhausen and Ruben, 1980), which is often used in fragment screening (Kerber et al., 2023) or for affinity determination (Williamson, 2013). The [ ${}^{15}$ N, ${}^{1}$ H]-HSQC of the KRAS (Kirsten rat sarcoma virus, mutant G13D) mutant was recorded in the absence and presence of the optical fiber (Fig. 2c), and we examined the peak shape by extracting the row and column slices (*slice* command in Topspin^®^). The peaks showed no significant difference in the presence or absence of the optical fiber inserted inside the probe (Fig. 2c). The two spectra were so similar that, in the spectra overlay (Fig. 2c), it is impossible to observe the spectrum of KRAS that was measured with the fiber (blue). Therefore, we conclude there is no need to remove the optical fiber once installed, as it does not interfere with the non-illuminated NMR experiments.

**Figure 3:**
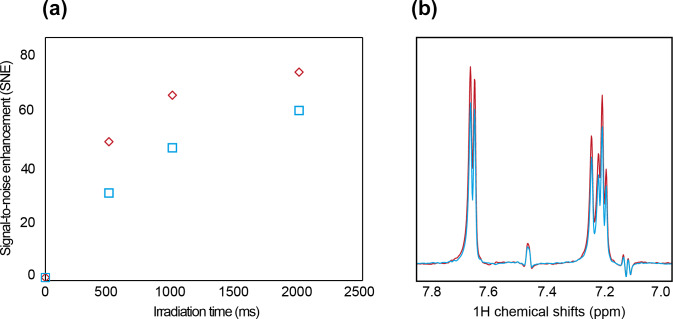
**(a)** SNE evolution with illumination time, with illumination from the top (red) and from the bottom (blue). **(b)** Signal intensity after 2 s of illumination from the top (red) and from the bottom (blue). The sample contained 30 µ M tryptophan and 10 µ M fluorescein, with oxygen-quenching enzymes, GOCAT, and glucose (see “Material and methods” section).

Finally, we assessed the photo-CIDNP NMR performances of our setup and compared them with the classic setup, where the optical fiber is coupled from the top of the NMR tube with the optical fiber dipped into the aqueous sample. The SNE is 74-fold and 60-fold when the sample is illuminated from the top and the bottom, respectively (Fig. 3). The difference is largely explained by the length of the optical path, i.e., the distance between the light entering the sample and the sample volume detected by the probe. Indeed, with the optical fiber inserted into the sample, the light path before reaching the measurement zone is approximately 5 mm, while when the optical fiber is inserted at the bottom the light path is approximately 10 mm. Using the Beer–Lambert law (Taniguchi et al., 2018; Dixon et al., 2005; Beer, 1852), and a molar absorption coefficient of approximately 11 000 (M cm) ${}^{-1}$ at a wavelength of 450 nm and a fluorescein concentration of 10 µ M, we estimated a transmission of 77.6 % after a 10 mm path length (bottom excitation) and 88.1 % after a 5 mm path length (top excitation). A second effect stems from Fresnel reflections (Beer, 1852) at the interfaces between fiber core to air, air to glass, and glass to water. The combined loss due to reflection was simulated to be 8.6 %.

On the contrary, inserting the fiber directly into the aqueous solution results in only 0.3 % Fresnel reflection due to the small difference in the index of refraction between the fiber core and the liquid. The combined effect of Fresnel reflection and absorption losses after a 10 mm path difference leads to power transmission of 71.1 % of the initial fiber power in the case of the excitation from the bottom and 87.7 % in the case of excitation of the immersed fiber (from the top). The ratio of the two transmitted powers for different setups, respectively for the bottom and from the top, is equal to 0.81 (0.711/0.877), which is in good agreement with the observed ratio of the differences in the photo-CIDNP signal of 60/74 = 0.81. Therefore, a simple way to improve the photo-CIDNP hyperpolarization could be to use higher laser powers to compensate for the optical density and the Fresnel reflection. However, this would yield more photosensitizer bleaching and should only be considered for applications with single or a low number of scans. Optimizing the photosensitized concentration can also improve the homogeneity of the illumination and maximize the photo-CIDNP hyperpolarization (Fig. S7). In both setups, detecting a low concentration of tryptophan (30 µ M) with a single-scan NMR experiment was possible. For comparison, in fragment screening with saturation transfer difference (STD) NMR screening, the ligand concentrations vary between 50 to 500 µ M, with a measurement time of 15–60 min. Boltzmann polarization NMR measurement time ranges from 15 min to 1 h, depending on the instrument's sensitivity and concentration. With photo-CIDNP fragment screening, we previously demonstrated the possibility of screening at concentrations as low as 5 µ M of ligand using single-scan experiments (Torres et al., 2023; Stadler et al., 2023), corresponding to 1–2 s. Recent advances in the ultralow concentration photo-CIDNP NMR measured tryptophan concentrations down to 20 nM (Yang et al., 2022); similar performances are expected to be possible with this setup as the light coupling is similar.

Current autosamplers can exchange a sample every 2–3 min as the sample undergoes a temperature equilibration (1–2 min), a lift up and down of the sample (10–20 s), and the automated shimming procedure (requires 30 s to 1 min). With the combination of photo-CIDNP hyperpolarized NMR experiments on the order of a few seconds and current commercial autosamplers, achieving a fully automated throughput of approximately 700 samples daily is possible. The implementation is straightforward and can likely be pushed by several times with optimization, fully automated. Irradiating from the bottom is also an easy solution for benchtop setups, which are often open on both sides.

## 3 Conclusion

The present work describes a simple way to introduce an optical fiber through a cryogenic probe's flow-cell accessory port to reach the near vicinity of NMR sample tubes. Such a port is accessible in all 5 mm standard CryoProbes (but not for 3 and 1.7 mm CryoProbes) and Prodigy CryoProbes from 300–900 MHz. This allows for the irradiation of the samples contained in NMR tubes from the bottom, taking advantage of the spherical bottom and resulting in a lensing effect. The light is, therefore, efficiently coupled with the sample tube, and a slight deviation in the positioning of the optical fiber is not critical. As the optical fiber can be maintained sufficiently far away from the measuring and shimming regions of the probe, the influence on field homogeneity is minimal. It does not affect the performance of the spectrometer. This is not surprising as others have successfully designed similar systems before us for room temperature probes, using optical fiber and quartz rods, which are more prominent (Tolstoy et al., 2009; Koeppe et al., 2011; Kuprov and Hore, 2004). Therefore, removing the optical fiber to perform other NMR experiments is unnecessary once installed. We verified that the photo-CIDNP performances were similar, and we could measure relatively low tryptophan concentrations with single-scan experiments. This design is the first to allow for high-throughput automated measurement of photo-CIDNP experiments as it is compatible with commercial autosamplers. It can be used equally for other light-coupled NMR experiments requiring high throughput. The presented setup will enable photo-CIDNP small-molecule screening to achieve its maximal throughput, corresponding to the throughput of the commercially available autosamplers, as photo-CIDNP fragment screening is measured with single-scan experiments (1–5 s). Such throughput is expected to increase with a faster autosampler, adapted workflow (temperature equilibration), and accelerated shimming procedures (Becker et al., 2022). Altogether, these advances will enable photo-CIDNP NMR small-molecule screening to achieve a throughput of >1500 samples daily, eventually up to 3000.

### Material and methods

For the optical simulations, Ansys Zemax OpticStudio was used in non-sequential mode. The software performs a Monte Carlo simulation by computing the path of many randomized light rays through the geometry modeled according to the experimental setup. The simulation accounted for the absorption of fluorescein at 10 µ M in water (0.10752 cm ${}^{-1}$ ), which was set to agree with the medium's optical density (OD), and the scattering was neglected as it is expected to have a minor influence.

We built an optical benchtop setup that illuminated 3 and 5 mm NMR tubes with lensed tube bottoms with multimode fibers of different NA and core diameters. The NMR tubes were coaxially held in standard Thorlabs 30 mm cage plates, while positioners were used to change the lateral and axial distances of the fiber with respect to the NMR tube. We monitored optically reflected powers with a fiber-based beam splitter on the entrance port and transmitted powers with a photodiode sensor on top of the (uncapped) NMR sample tube. It was also possible to detect power levels at different axial positions within the liquid sample using an immersed photodiode combined with an external current–voltage converter (Fig. S6).

The fiber is inserted from the entry of the air conduct, which is typically connected to the Bruker cooling unit (BCU). The connection to the BCU is maintained using a T-connector to accommodate the optical fiber and the BCU tubing. The fiber is inserted until the user feels the resistance of the NMR tube; then the optical fiber is pulled down by a few millimeters.

All NMR measurements were performed at 298 K on a Bruker Avance III HD 600 MHz spectrometer equipped with a Bruker TCI 600 ${}^{1}$ H/ ${}^{13}$ C/ ${}^{15}$ N MHz CryoProbe. The laser used was a Thorlabs L450P1600MM, which is a diode laser emitting at 450 nm. The laser light was coupled (using appropriate coupling optics) into an optical fiber (Thorlabs, FG950UEC) with a length of 10 m and a diameter of 0.4 mm. The light power output by the laser diode is 1.6 W, and the light power measured at the optical fiber output is 1.0 W due to loss during laser diode–optical fiber coupling.

Photo-CIDNP experiments were performed at 30 µ M tryptophan concentration in 100 mM potassium phosphate buffer at pH = 7.2, GOCAT enzyme with glucose as described herein, and 10 µ M fluorescein. To prevent photosensitizer quenching, the enzyme cocktail glucose oxidase (GO, 120 kDa), catalase (CAT, 240 kDa), and d-glucose (G, 180 Da) were used at a concentration of 200 nM, 140 nM, and 2.5 mM, respectively. The stock solutions were 4.0 µ M for GO and 4.0 µ M for CAT, respectively, in 10 mM NaPO ${}_{4}$ buffer and pH = 7.2. The glucose stock solution was 500 mM in D ${}_{2}$ O with 0.02 % NaN ${}_{3}$ .

The reference sample from Bruker was used for the line shape evaluation, i.e., 0.3 % CHCl ${}_{3}$ in acetone-d ${}_{6}$ .

The [ ${}^{15}$ N, ${}^{1}$ H]-HSQC spectrum was measured at 600 MHz ${}^{1}$ H NMR frequency with 600 µ M ${}^{15}$ N-labeled KRAS in 20 mM HEPES, 100 mM NaCl, 5 mM MgCl ${}_{2}$ , and 2 mM TCEP, at pH = 7.4 and temperature of 298 K. Typically, 256 ( $t_{1,max}$ ( ${}^{15}$ N) = 47.6 ms) × 2048 ( $t_{2,max}$ ( ${}^{1}$ H) = 60.8 ms) complex points, an inter-scan delay of 0.8 s, and 16 scans per increment were measured. The data were zero-filled to 4096 points in the direct proton dimension and 512 points in the ${}^{15}$ N dimension. Processing was done with a shifted cosine window function for both dimensions.

## Supplement

10.5194/mr-5-61-2024-supplement The supplement related to this article is available online at: https://doi.org/10.5194/mr-5-61-2024-supplement.

## Supplement

10.5194/mr-5-61-2024-supplement
10.5194/mr-5-61-2024-supplement The supplement related to this article is available online at: https://doi.org/10.5194/mr-5-61-2024-supplement.

## Data Availability

All the data are available upon request to one or the corresponding authors.

## Appendix

The supplement related to this article is available online at: https://doi.org/10.5194/mr-5-61-2024-supplement.
