# Supplementary material for: Light-coupled cryogenic probes to detect low-micromolar samples and allow for an automated NMR platform
Source: Magn Reson (Gott). 2024 May 15;5(1):61–7. doi: 10.5194/mr-5-61-2024 (PMC12082487; doi:10.5194/mr-5-61-2024)
Supplement: The supplement related to this article is available online at: https://doi.org/10.5194/mr-5-61-2024-supplement. [file mr-5-61-2024-supplement.pdf]

Supplement of Magn. Reson., 5, 61–67, 2024
https://doi.org/10.5194/mr-5-61-2024-supplement
© Author(s) 2024. CC BY 4.0 License.

Open Access
MAGNETIC
RESONANCE

Supplement of

Light-coupled cryogenic probes to detect low-micromolar samples and allow for an automated NMR platform

Wolf Wüster et al.

Correspondence to: Roland P. Riek (roland.riek@phys.chem.ethz.ch) and Felix Torres (ftorres@nexmr.com)

The copyright of individual parts of the supplement might differ from the article licence.

Contact: roland.riek@phys.chem.ethz.ch; ftorres@nexmr.com

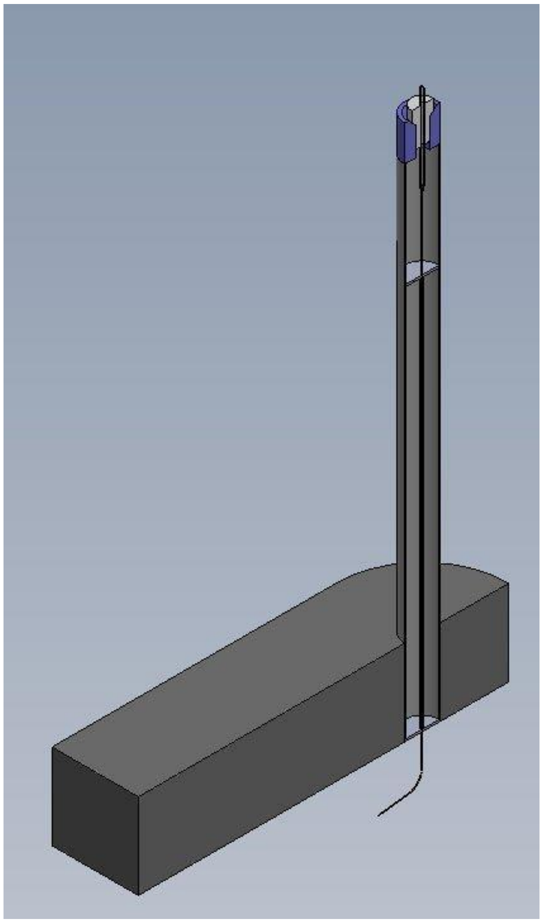

Figure S1: CAD drawing of a Bruker CryoProbe with an optical fiber inserted via the bottom flow-accessory port.

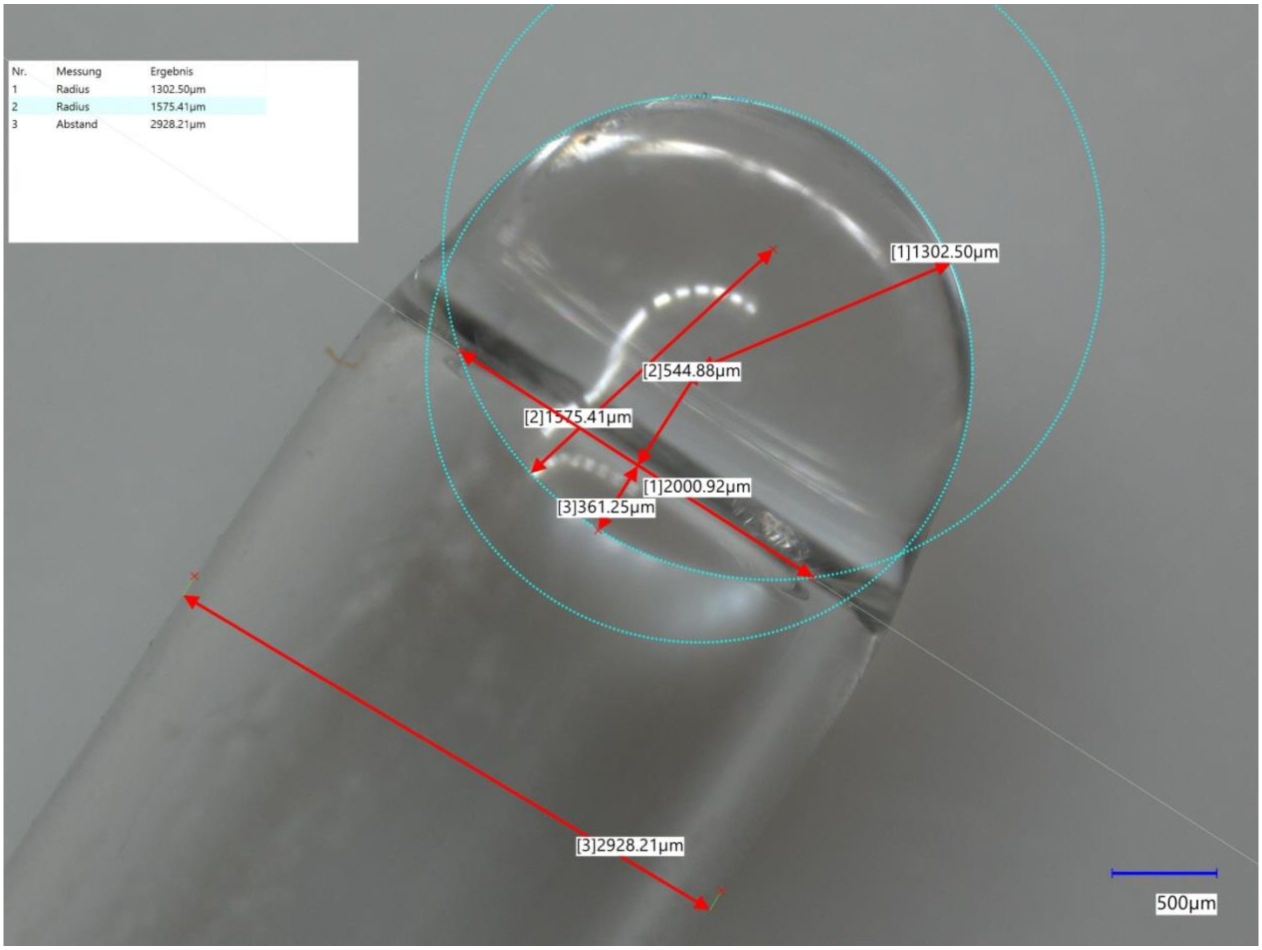

Figure S2: Microscopic photograph of a 3mm NMR tube with lensed bottom. This lens shape is usually present in standard tubes. The two circles define the two curvatures of the convex lens.

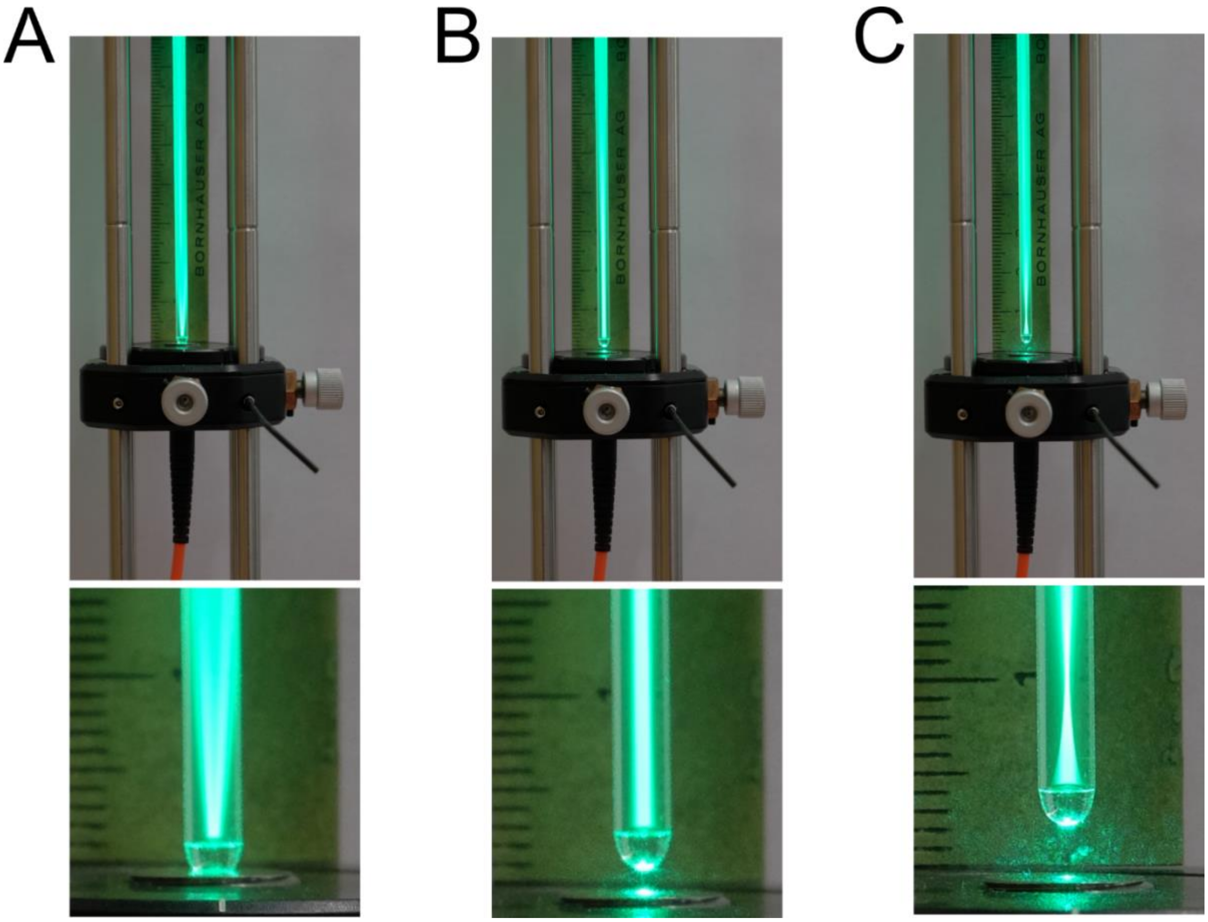

Figure S3: Benchtop experiment with a fiber NA=0.22 and a 3mm NMR tube. The fiber is positioned at different distances along the z-axis from the NMR tube. In all cases the light is guided within the sample liquid and NMR glass tube walls via total internal reflection (top part of the figure) Depending on the offset distance, the beam has different shapes after entering the liquid sample (bottom part of the figure: zoom-in of the top around the light entrance). A) Offset: 0.0 mm – the beam is initially divergent B) Offset: 1.0 mm – the beam is initially collimated C) 2.5 mm – the beam is initially focused.

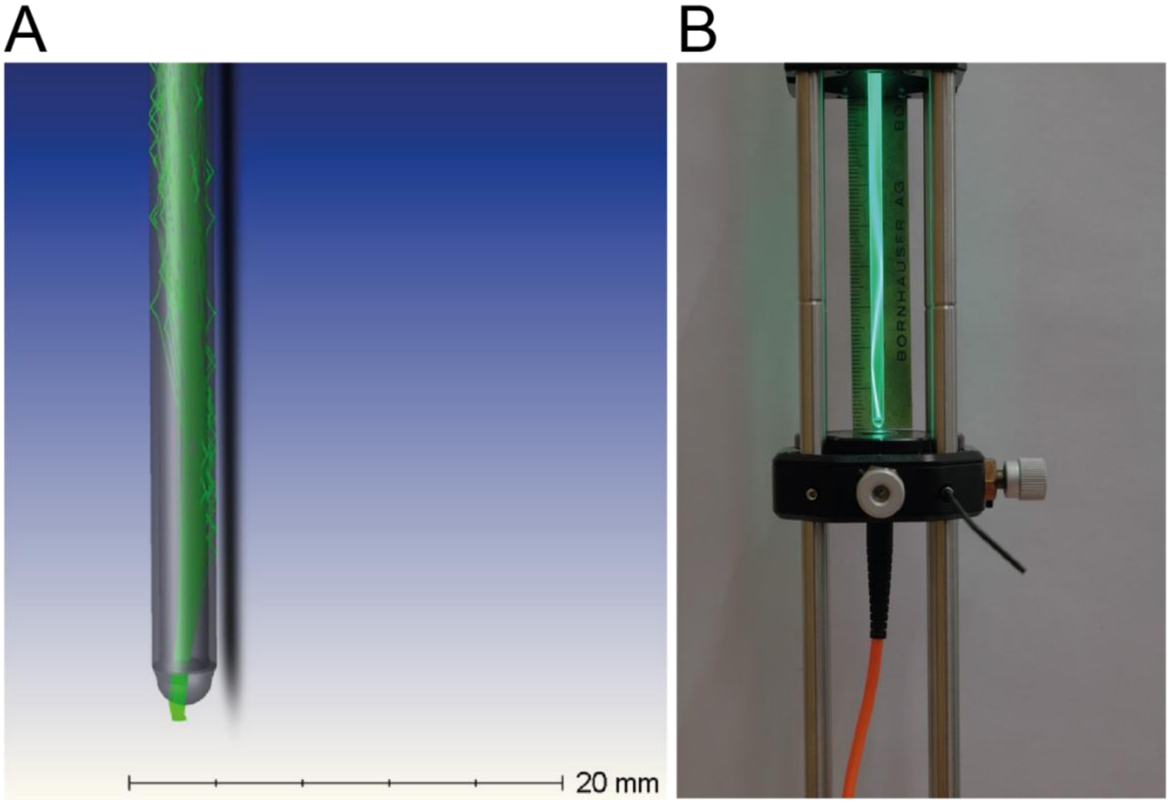

Figure S4: A) Monte Carlo simulation of diffracted rays into the sample volume. The rays exit the fiber in a light cone defined by the fiber NA (0.22) with a y-axis offset of 0.2 mm. B) Benchtop experiment with a fiber NA=0.22, a 3mm NMR tube, and a transverse offset of 0.5 mm. In agreement with the simulation, the light beam is reflected at the sample walls via total internal reflection.

The optic fiber-coupled cryogenic probes are also compatible with standard 5mm tubes. Figure S5 shows a 5 mm tube illuminated with an NA=0.22 fiber for two longitudinal offsets. The working principle is the same. Depending on the longitudinal offset, the homogeneity of the light in the NMR active region can be optimized.

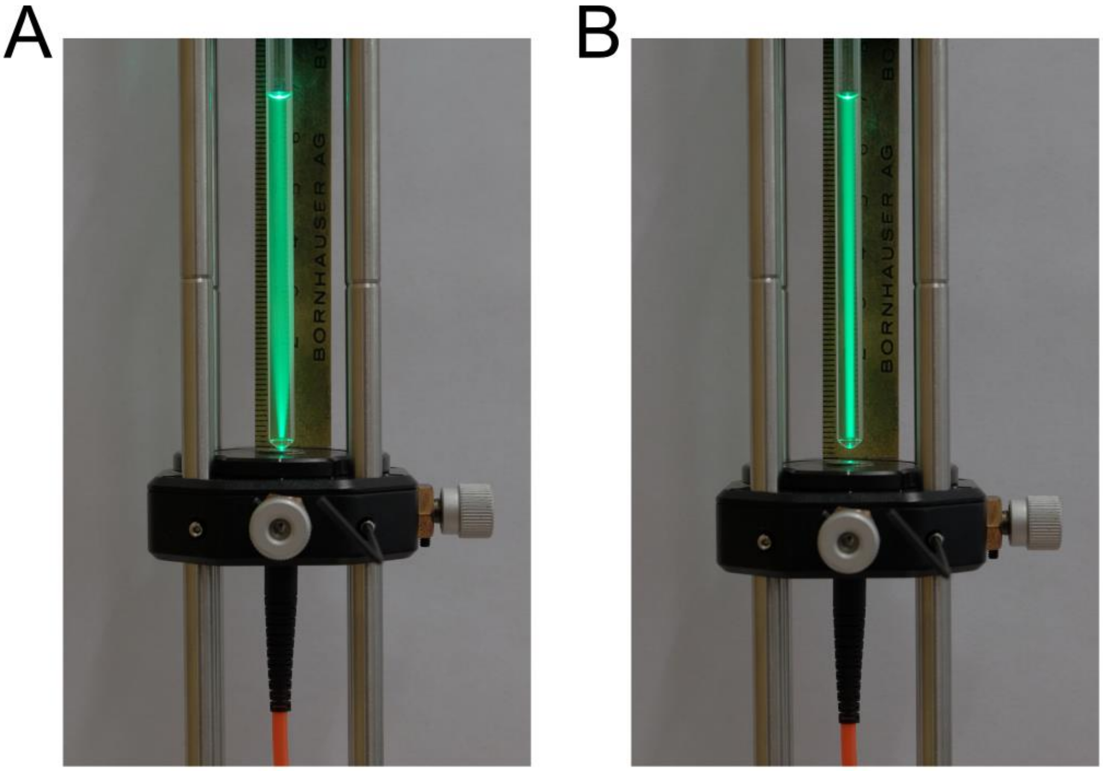

Figure S5: Optical Bench Top experiment of a 5mm NMR tube with lensed bottom illuminated by a NA=0.22 optical multimode fiber. A) Longitudinal offset: 0 mm. Here the beam is initially divergent and the light in the NMR active region illuminates the whole sample volume. B) Longitudinal offset: 2.5 mm. Here, the light beam is collimated, and only part of the sample volume is illuminated.

To get an experimental estimate of the absorbed optical power as a function of the position in the liquid sample, we immersed a photodiode inside the sample for four different fluorescein concentrations. We used the recorded power levels at 0 µM (distilled water) as a reference value and normalized all powers to these reference powers; see Figure S6.

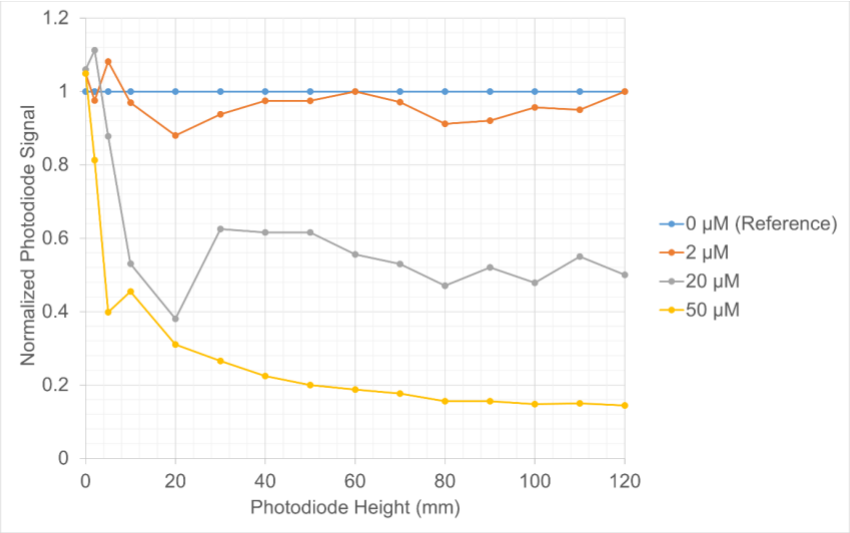

Figure S6: Normalized power level on photodetector as a function of height in the NMR liquid sample tube. The position of 0 mm corresponds to the photodiode at the bottom of the tube. The Fluorescein concentrations vary between 0 µM and 20 µM, and the power levels remain high (approximately 60%) for different heights of the photodiode position. For high concentrations of 50 µM and higher, we see a significant drop in the power levels due to high light absorption in the sample.

In Figure S7 we calculated the z-position-dependent absorbed light within the NMR sample. We find that for fluorescein concentrations around 20 µM, we can expect the largest photo-CIDNP signal, which will drop towards lower and higher concentrations.

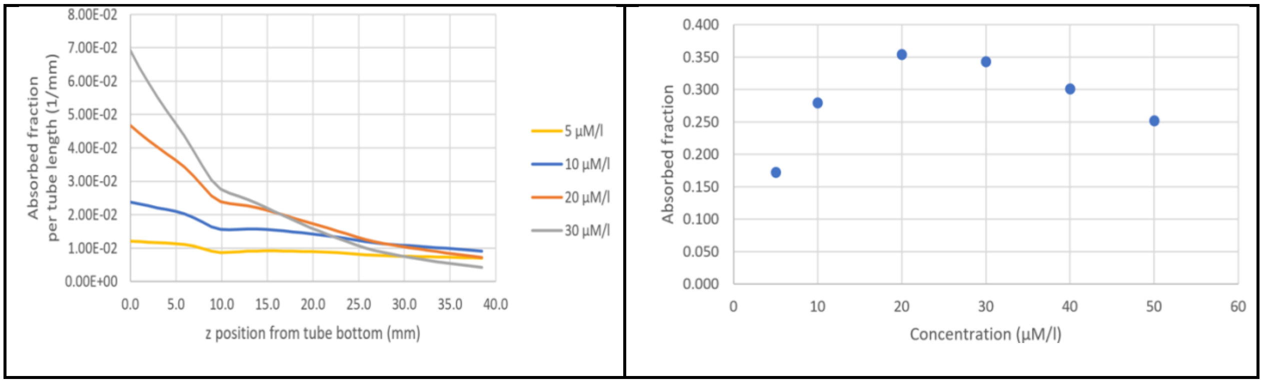

Figure S7 Left: Simulation of the absorbed light by the sample per tube length for different fluorescein concentrations. Right: Absorbed light within the NMR active volume vs. Fluoreceine concentration. At 20 µM concentration, most light is absorbed, but the sample volume is illuminated more uniformly at lower concentrations. Tube diameter: 3 mm, Fiber distance to tube: 2 mm, Fiber NA: 0.22, Fiber diameter: 0.9 mm.
